# Fluorescence-Based Microendoscopic Sensing System for Minimally Invasive In Vivo Bladder Cancer Diagnosis

**DOI:** 10.3390/bios12080631

**Published:** 2022-08-11

**Authors:** Sanghwa Lee, Jeongmin Oh, Minju Cho, Jun Ki Kim

**Affiliations:** 1 Biomedical Engineering Research Center, Asan Medical Center, Asan Institute for Life Science, Seoul 05505, Korea; 2 Department of Convergence Medicine, University of Ulsan College of Medicine, Seoul 05505, Korea

**Keywords:** 5-aminolevulinic acid, bladder cancer, ferrochelatase staining, microendoscopy, minimally invasive diagnosis, protoporphyrin IX

## Abstract

Bladder cancer is commonly diagnosed by evaluating the tissue morphology through cystoscopy, and tumor resection is used as the primary treatment approach. However, these methods are limited by lesion site specificity and resection margin, and can thereby fail to detect cancer lesions at early stages. Nevertheless, rapid diagnosis without biopsy may be possible through fluorescence sensing. Herein, we describe a minimally invasive imaging system capable of sensing even small tumors through a 1.2 mm diameter flexible fiber bundle microprobe. We demonstrate that this new device can be used for the early diagnosis of bladder cancer in rats. Bladder cancer was induced in rats using the carcinogen *N*-butyl-*N*-(4-hydroxybutyl)nitrosamine (BBN), and a togglable filter capable of PpIX fluorescence sensing was installed in the microendoscopic system. Following 5-aminolevulinic acid administration, tissue in the early stages of bladder cancer was successfully identified with fluorescence detection and confirmed with hematoxylin/eosin and ferrochelatase staining. Although the time required for BBN to induce bladder cancer varied between 3 and 4 weeks among the rats, the microendoscopic system allowed the minimally invasive follow-up on cancer development.

## 1. Introduction

Optical imaging technology should enable a rapid histological diagnosis (as an optical biopsy), but also increase diagnostic sensitivity through the margin evaluation and tissue biopsy of a suspected lesion (focusing on a target site), thereby avoiding unnecessary risks, high costs, and the requirement for polypectomy [1,2]. Therefore, cystoscopy-based signal sensing technology has attracted significant attention for obtaining real-time biometric information for bladder cancer resection. Currently, this approach is vital for diagnosing bladder cancer, because it allows for the comprehensive examination of the entire bladder and urethra, including the prostate urethra. Nevertheless, bladder cancer is diagnosed by observing the surface morphology of the bladder through cystoscopy, and bladder tumor resection is the primary treatment method. Modern urologic endoscopy technologies, such as photodynamic diagnosis (PDD), narrow-band imaging, and optical coherence tomography, have been developed to overcome some of the limitations of the current methods. Diagnostic challenges that these new methods are expected to resolve include early-stage tumors not showing surface morphology changes, mid-stage tumors being obscured by folds and textures in the bladder epithelium, and satellite tumors being overlooked during resection [3]. Due to these challenges, a diagnosis error is more likely to occur if a tumor has to be diagnosed using only white-light endoscopy, rather than with techniques which include additional fluorescence imaging. Overcoming the limitations of brightfield endoscopy would, ultimately, support enhanced treatments and improve patient outcomes [4]. Moreover, state-of-the-art devices for cystoscopy in rodents have limited field of view, are not often used for fluorescence imaging, and are not geometrically suitable for translation to the clinic [5,6]. As a result, some authors have had pessimistic evaluations of the potential of ultrathin cystoscopes for the diagnosis and tracking of tumor development in orthotopic models of bladder cancer [7].

Cancer fluorescence imaging using 5-aminolevulinic acid (5-ALA) has recently been approved by the U.S. Food and Drug Administration [8] and has gathered significant attention for early diagnosis and obtaining optimal PDD results. 5-ALA is a precursor of porphyrin, heme, and bile pigments, and is metabolized into protoporphyrin IX (PpIX) for heme synthesis. The fluorescent PpIX is synthesized at higher rates and is less actively metabolized by tumors; thus, it accumulates within tumor cells, making it a promising fluorescent marker for tumor detection. PpIX can be used as a contrast agent in endoscopy for the clinical imaging of brain, bladder, and gastrointestinal cancers [9,10], as well as for molecular imaging in cancer surgery [11].

To date, treatment options for bladder cancer have been developed to overcome the patient burden and have been targeted to the specifics of the disease [12,13]. For testing the performance of chemotherapeutics, animal models have been designed with bladder cancer induced with N-butyl-N-(4-hydroxybutyl)nitrosamine (BBN)-based carcinogens. These animal models mimic muscle-invasive human carcinomas [14,15,16]. Nonetheless, even if consistent dosages of carcinogens are supplied, minimally invasive in vivo monitoring of targeted therapeutics is difficult because of the timing and size of the cancerous lesions, as well as their location in the bladder, which varies between patients.

**Figure 1:**
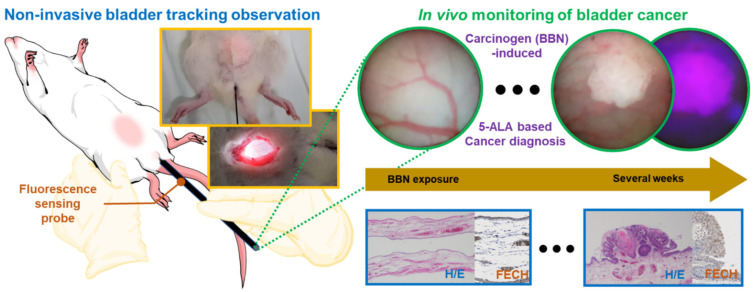
Schematic diagram and images of the experimental minimally invasive monitoring procedure and fluorescence imaging of BBN-induced bladder cancer using a flexible fluorescence imaging microprobe. 5-ALA, 5-aminolevulinic acid; BBN, N-butyl-N-(4-hydroxybutyl)nitrosamine; H/E, hematoxylin/eosin staining; FECH, ferrochelatase staining.

In this study, we describe a novel, minimally invasive cancer-monitoring system that uses a 1.2 mm flexible microscopic endoscope for in vivo imaging of bladder cancer (Figure 1). Compared to previously demonstrated state-of-the-art small animal cystoscopes, the endoscope presented is geometrically suitable for both minimally invasive small animal bladder evaluations and for transition to the clinic, and has a wider field of view. Moreover, the demonstrated approach takes advantage of the early diagnosis potential of 5-ALA as a fluorescence contrast agent. A successful demonstration of the PDD fluorescence modality in an ultrathin cystoscope has the potential to rehabilitate cystoscopy for tracking tumor development in rodent bladder cancer models, and the 1.2 mm microscopic endoscope system is also suitable for fluorescence sensing following insertion through the biopsy channel of a general cystoscope currently used in clinical practice.

## 2. Materials and Methods

### 2.1. Animal Preparation for BBN-Induced Bladder Cancer

Under the laws of the Republic of Korea, all following animal experiments were approved by the Institutional Animal Care and Use Committee (IACUC) of the Asan Institute for Life Sciences, Asan Medical Center (approval no. 2020-12-111). Six-week-old female Sprague Dawley rats (Orientbio, Seongnam, Korea) were randomly divided into two groups: a bladder cancer group, wherein the mice were provided 0.1% BBN (Tokyo Chemical Industry, Tokyo, Japan) dissolved in the drinking water (protected from light) for 4–6 months, and a control group. Each water container was replenished according to the consumption rate of the corresponding cage. Eight rats with BBN-induced bladder cancer and three healthy rats were maintained together and observed for the same period. Brightfield and fluorescence images were acquired three, six, nine, twelve, fifteen, and eighteen weeks after BBN supply. Control images were acquired one week before BBN supply.

### 2.2. Preparation of the Microendoscopy-Based Fluorescence Sensing System

**Figure 2:**
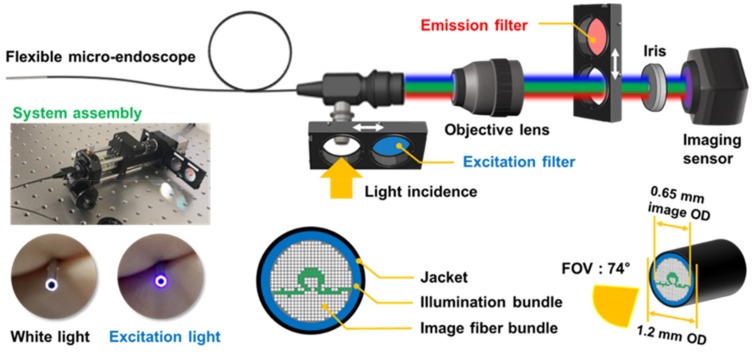
Optical structure and dimensions of the microendoscopy-based fluorescence sensing device.

A schematic illustration of the microendoscopic system is presented in Figure 2. An endoscope with a 30,000-pixel fiber bundle as its core surrounded by an illumination fiber bundle and covered with a ring-shaped jacket was used as a custom product (FIGH-30-650S; Myriad Fiber Imaging, Dudley, MA, USA). The field of view (FOV) of images acquired from the endoscope head was 74°, the total outer diameter of the endoscope was 1.2 mm, and the core fiber bundle for imaging had an outer diameter of 0.65 mm. The image of the FOV area formed by the lens of the endoscope head was transmitted to the camera through the core fiber bundle, and the inside of the whole bladder of the rat was scanned and stored as a video of three to four minutes in length.

A multi-LED lamp (X-cite Xylis; Exelitas, Mississauga, ON, Canada) with a wavelength range of 360–770 nm was connected as a light source for brightfield and fluorescence excitation and the light intensity could be adjusted in 1% increments from 5% to 100%. The intensity was set at approximately 10–25% and 80–100% for obtaining brightfield and fluorescence images, respectively. The maximum (100%) light output from the microprobe head in Figure 2 corresponded to 13.5 mW optical power, as measured with a photodiode power sensor (S130C; Thorlabs, Newton, NJ, USA), based on the assumption of a spectral maximum at 635 nm.

The fluorescent signal was acquired by switching in a filter (FBH 400-40; Thorlabs, Newton, NJ, USA) for the excitation light source in the 360–440 nm range and an emission filter (FBH 650-40; Thorlabs) to selectively acquire only the fluorescent signal from the target tissue, which was in the 610–690 nm wavelength range. A 10× objective lens (Plan N; Olympus, Tokyo, Japan) was used to magnify the 0.65 mm diameter image of the fiber bundle, which was transmitted to a 1920 × 1080-pixel CMOS OV2710 imaging sensor (ELP-USB; Shenzhen Ailipu Technology Co., Shenzhen, China). The objective lens was mounted on a translation stage (SM1Z; Thorlabs) to control the focal length, a 650 nm long-pass filter installed in the camera was removed before assembly, and the imaging performance was improved by installing an iris (SM1D12C; Thorlabs) in front of the imaging sensor. The image of fluorescence generated by the sample was transmitted from the proximal tip of the fiber bundle to the camera at 10× magnification by the objective lens. The transmittance of the acylate fiber used in the image bundle was 99.8%, and the OV2710 camera sensing chip used in this system detected light of 15 lux or more. However, the fluorescence generated and detected from tumors in the bladder was also affected by the distance between the endoscope head and the subject and the scattering and absorption of light due to residual urine. Fluorescence sensing optimization was performed by adjusting the distance from the living tissue.

### 2.3. 5-ALA-Induced Fluorescence Imaging

5-ALA (Tokyo Chemical Industry) was dissolved in phosphate-buffered saline-HCl solution (pH 2.0; Asan Medical Center, Korea) to a final concentration of 400 mg/mL. 5-ALA (100 μL/100 g in solution) was administrated to the rats by oral gavage 4 h before fluorescence imaging according to body weight (400 mg/kg). Subsequently, the rats were anesthetized with 30 mg/kg tiletamine/zolazepam (Zoletile; Virbac, Carros, France) and 5 mg/kg xylazine (Rompun; Bayer, Leverkusen, Germany). The depth of anesthetization was evaluated by foot pinching. Intravenous Angiocath 24Ga catheter (BD, Franklin Lakes, NJ, USA) was inserted in the urethra. The bladder was then emptied by applying pressure. Phosphate-buffered saline (PBS) heated to 37 °C was injected through the intravenous catheter and emptied to wash the inner bladder wall. The washing process was repeated thrice until the bladder was cleared. The intravenous catheter was pulled out, and a 1.2 mm diameter fiberscope was inserted through the urethra. The inner surface of the bladder was inspected with the fiberscope and attached optical system.

### 2.4. Hematoxylin/Eosin and Ferrochelatase (FECH) Staining for Bladder Cancer Histopathology

After six months of BBN administration, rats in deep anesthesia were euthanized by bloodletting. The bladder was collected and fixed in 4% paraformaldehyde solution for over 24 h. Fixed bladders were embedded into paraffin blocks and sectioned in 3 µm thick slices, which were then stained with hematoxylin/eosin for morphological evaluation. Immunohistochemistry was performed manually (OBEN Bio, Suwon, Korea) using a primary anti-FECH rabbit antibody (1:100 dilution; LSBIO, Seattle, WA, USA). The stained tissue sections were visualized using a visible-light microscope (N-800M; Novel Optics, Nanjing, China) coupled with a 5.0 MP digital camera (HDCE-X5N; Novel Optics). The obtained images were composited and saved as MView MRXS files.

## 3. Results

### 3.1. Minimally Invasive Brightfield and Fluorescence Images of Bladder Cancer In Vivo

**Figure 3:**
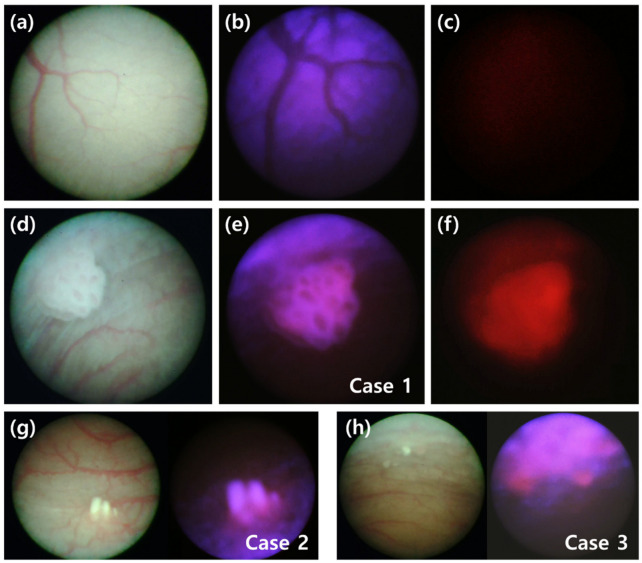
Minimally invasive in vivo brightfield and fluorescence images of bladder in rats. (**a**–**c**) The inside of a healthy bladder, imaged with (**a**) brightfield illumination, (**b**) excitation filter only, and (**c**) with an excitation filter and emission filter both inserted. (**d**–**f**) Images of the inside of a bladder with cancerous lesions taken with (**d**) brightfield illumination, (**e**) excitation-filtered illumination, and (**f**) both excitation and emission filters at week 13 following BBN treatment (Case 1). (**g**,**h**) Cases 2 and 3 show additional cases of bladder cancer in other rats.

To review the performance of the microendoscopy-based brightfield and fluorescence sensing system, the difference in the intravesical images of rats with BBN-induced bladder cancer and of healthy rats was investigated, and representative images are shown in Figure 3. The brightfield images of the blood vessel pattern in the normal rat bladder (Figure 3a) appeared negative under a light source with an excitation wavelength of 360–440 nm (Figure 3b). The wavelength of the excitation light was matched with that of the absorption band of the blood to visualize the violet color reflected from the bladder wall. The reflected light was also blocked by inserting the emission filter in front of the imaging sensor (Figure 3c). A brightfield image of a BBN-fed rat demonstrating well-developed cancer tissues at week 13 is shown in Figure 3d. As shown in Figure 3e (Case 1), injecting excitation light induced red light fluorescence in the cancer tissues. Inserting the emission filter aided in visualizing the fluorescence intensity in these cancer tissues (Figure 3f). As shown in Figure 3g,h, fluorescence in bladder cancer was localized within cancer tissue (Case 2) even when cancer tissues were widely distributed in the bladder wall (Case 3), confirming the significance of imaging using PpIX fluorescence.

### 3.2. Early Diagnosis of Bladder Cancer via Fluorescence Sensing Follow-Up

**Figure 4:**
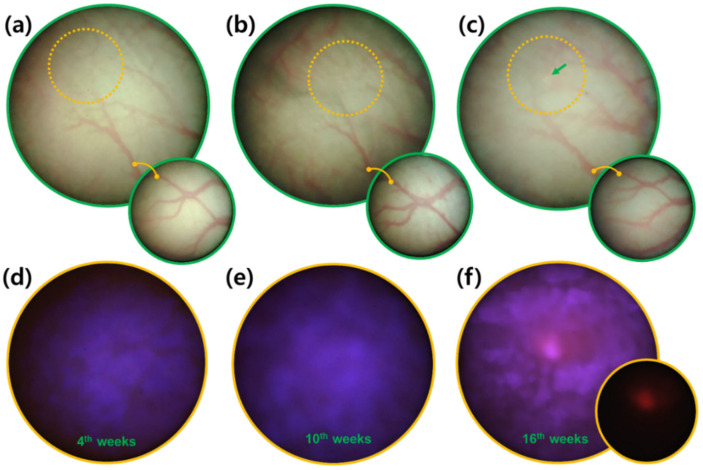
Tracking and monitoring of bladder cancer development. Brightfield and fluorescence images were obtained from the same bladder tissue locations at weeks (**a**,**d**) 4, (**b**,**e**) 10, and (**c**,**f**) 16 of BBN exposure. Orange circles in (**a**–**c**) brightfield images denote the approximate corresponding positions in (**d**–**f**) fluorescence identification based on the vascular patterns. The green arrow indicates the location of a cancer lesion. The shape of the major blood vessels is highlighted in the small lower circles in (**a**–**c**). The fluorescent signal (red) of the cancer tissue is highlighted in the small lower circle in (**f**).

Although the same concentration of BBN was supplied in the drinking water of each rat, the time at which cancer lesions were visible varied among the experimental animals. For example, in one rat, the initial fluorescent signal from bladder cancer tissues was detected at week 7 upon BBN administration, whereas in another rat, the initial fluorescence was only detected at week 19. Since these two cases were observed at 3-week intervals, there was a difference in the time of cancer occurrence of up to 15 weeks (Figure 4). Despite these differences, the new microendoscopy-based brightfield and fluorescence sensing system exhibited a robust capacity to detect cancerous tissues even during early tumor development (Figure 4). Hence, it may overcome the limitations of judgment on tumor localization through the surface morphology of brightfield information. Nevertheless, while the inside of the rat bladder was scanned and recorded with brightfield and excitation light (Figure 4), the localization of cancer tissue was confirmed with fluorescence (Figure 4f, small circle). In Figure 3 above, the verification of the imaging system was performed on a tumor that could be easily seen in the brightfield, demonstrating that the fluorescence of morphologically distinct tumor tissue was clearly distinguishable from the minor autofluorescence of the bladder epithelial layer. Figure 4 demonstrates the successful fluorescence sensing of an early-stage tumor that was not easily visible in the brightfield.

Finding the exact location in the scan images of the bladder at a time point before the detection of the cancer fluorescent signal was possible by following the same blood vessel pattern (Figure 4a–c). Therefore, specifying the time and location of cancer occurrence in the same individual was possible. These results demonstrated that this fluorescence detection sensing system could be easily applied to monitor the in vivo effects of new drugs and treatment strategies for bladder cancer.

### 3.3. Tissue Biopsy Validation of Fluorometric Bladder Cancer Images

**Figure 5:**
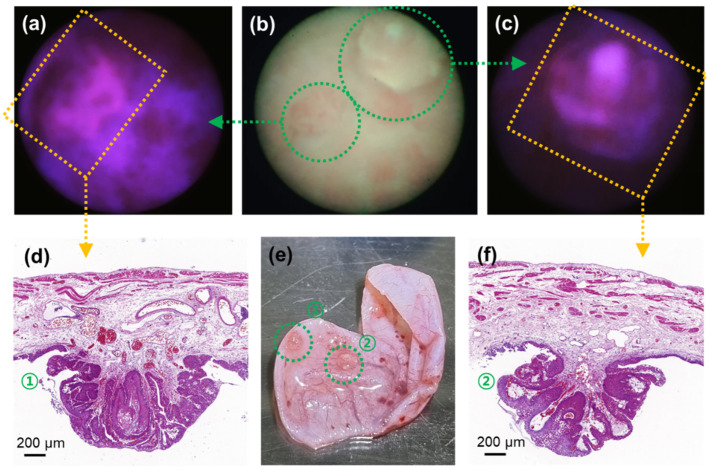
Biopsy process and H/E staining results of bladder cancer tissue at week 10 observed with brightfield and fluorescence imaging. (**a**,**c**) Two neighboring cancer lesions observed with fluorescence imaging and (**b**) corresponding brightfield image. (**d**,**f**) H/E staining results of the excised bladder cancer tissues. (**e**) Image of the excised bladder with the cancer lesions (highlighted in green dotted circles).

To confirm the specificity of this detection tool, the cancer tissue identified from the fluorescence sensing system in the rat bladder was extracted and evaluated further (Figure 5). The cancer lesions were easily identified with the naked eye (Figure 5e) and were positively detected with brightfield (Figure 5b) and fluorescence (Figure 5a,c) images. Since the diameter of the image fiber bundle was 0.65 mm, it was possible to estimate the diameter of the sample through ultra-close-up imaging, which revealed that the diameters of the lesions were approximately 1.62 and 1.58 mm. Notably, given that the field of view of this microendoscopic system was 74°, the size of the tumors could vary depending on the distance, which could not be controlled; thus, it could be difficult to accurately measure the size. Nonetheless, since the distance at which the brightfield image was recognizable was within 5 mm, an area with a diameter of ≥7 mm could be observed.

The cross-sectional analysis of these cancer lesions was further visualized with hematoxylin/eosin (H/E) staining (Figure 5d,f). For example, the tissue in Figure 5d was confirmed to be a BBN-induced cancerous tissue by its keratinization level [17], differences in nuclear and cytoplasmic volumes [18], and the empty staining and overstaining of the nuclear region [19]. In addition, the tissue in Figure 5f was confirmed to be bladder cancer by the presence of numerous irregular cell shapes and internal lining of unknown layers in the cell mass [18]. Therefore, the microendoscopy-based imaging and sensing system could be used to effectively and precisely detect cancerous tissues in the bladder.

### 3.4. Feasibility of Fluorescence Sensing Diagnosis

**Figure 6:**
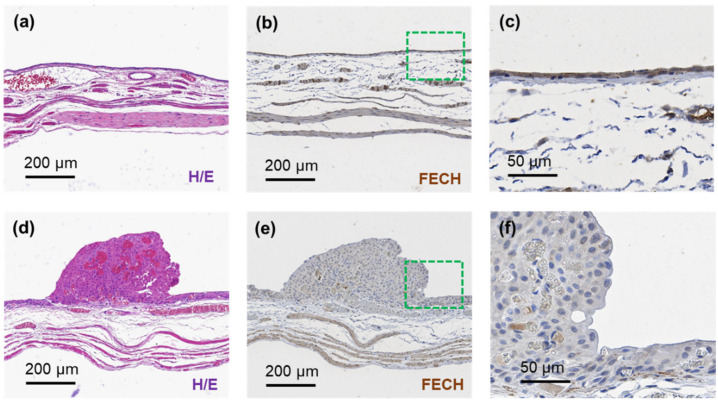
(**a**) H/E and (**b**) FECH staining of healthy bladder tissue, and (**c**) magnified image of the green dotted line in (**b**). (**d**) H/E and (**e**) FECH staining of a bladder cancer lesion, and (**f**) magnified image of the square dotted line in (**e**).

To confirm that the bladder cancer-specific fluorescence was accurately detected, the cancerous lesions were stained with ferrochelatase (FECH). When 5-ALA was orally administered to animals with cancer, the phosphor compound PpIX was not metabolized in the tumor; instead, it accumulated and fluoresced. Moreover, it is widely recognized that FECH expression is strongly related to PpIX accumulation, with its absence resulting in lower PpIX metabolism [20,21]. Herein, FECH density was measured in fluorescence-measured cancer tissues and compared with that of control samples (Figure 6). The H/E staining of healthy bladder tissue samples showed that the epithelial layer was evenly distributed with approximately 5–7 cells, and was separated from the muscle layer and the stromal layer (Figure 6a) [22]. Moreover, the signal of the FECH signal was mainly detected in the epithelial layer (Figure 6b,c) [21]. In contrast, the analysis of a tissue cross-section of a protruding cancer lesion showed that the epithelial layer was significantly thickened, and the cancerous cells had larger nuclei (Figure 6d) [18]. Moreover, the cancer cell mass had visible blood vessels (Figure 6d,e) [23]. Unlike in normal tissues, the FECH-stained area in the cancer cell mass was negligible (Figure 6e,f). Collectively, these results confirmed the feasibility of using fluorescence sensing for bladder cancer tissue detection in vivo.

## 4. Discussion

The strength of PpIX-based PDD lies in its enhanced tumor sensitivity. In human clinical trials, the application of 5-ALA or hexyl-ALA ester (HAL) for bladder carcinoma PDD has increased tumor detection rates from 77% (brightfield) to 95–97% and 97%, respectively [24,25,26]. PDD also allows for earlier tumor detection and treatment, improving patient outcomes. In this study, minimally invasive fluorescence-based in vivo cancer detection was actualized by integrating imaging and illumination with a small (1.2 mm diameter) fiber bundle suitable for transurethral access to the bladder of small animals. Monitoring induced PpIX accumulation and comparing it with the FECH staining of bladder cancer tissues enabled the precise detection of cancer lesions in the bladder even at the early stages of cancer development. Due to its narrow diameter and modular design, this flexible microscopic endoscope system could be directly applied in the biopsy channels of commercially available endoscopes, which typically have diameters of approximately 2.8 mm.

Previous studies incorporating in vivo fluorescence endoscopy for bladder cancer detection in rats used xenograft or orthotopic tumor models [27,28]. Unlike these models, BBN causes tumorigenesis without surgery. Tracking BBN-induced tumors is complicated by the stochastic nature of tumor development and because the tumor location is unknown in advance. In agreement with a previous study [29], we found a considerable variation in the BBN-induced bladder cancer model, with up to a 15-week variation in tumorigenesis time. Therefore, BBN studies typically rely on detecting large tumor masses [30] or ex vivo fluorescence imaging [31]. The device presented in this study overcame this significant limitation of BBN-based tumor studies by enabling the minimally invasive yet sensitive detection of early tumor fluorescence.

Although the present study had many parallels to prior cancer-targeted small animal cystoscopies, the unique combination of a miniaturized probe and PpIX fluorescence imaging enabled an early diagnosis, which was not possible in previous studies. Minimally invasive transurethral cancer diagnosis using PpIX in rats was previously reported. However, such studies were limited to late-stage tumors, brightfield endoscopy, nonimaging optical diagnoses, or ex vivo analyses [30,32,33]. Therefore, this study was differentiated by having achieved the early detection of bladder tumors in small animals via minimally invasive in vivo PpIX-based sensing. To the authors’ best knowledge, a minimally invasive fluorescence microendoscope has not previously been used to detect early-stage bladder cancer in rats in vivo.

Nonetheless, the system used in this study had some limitations. The resolution was constrained by the pitch (fiber density) of the flexible fiber bundle. Although the resolution of the sensing camera was 2 megapixels (1920 pixels × 1080 pixels), the resolution of the displayed image depended on the fiber bundle resolution, which was limited by the present fiber to 30,000 pixels. The resolution of each image was dependent upon the distance from the fiber tip to the tissue, with the highest resolution limited by the fiber pixel spacing of 3.3 µm. While this may be improved with multicore or structured-core optical fiber bundles, fluorescence detection is also constrained by the numerical aperture of the probe; thus, inherent tradeoffs exist between fluorescence sensitivity, resolution, and fiber core size. A significant general limitation for in situ cystoscopic PDD is low specificity, with increased chances of false-positive results owing to tissue inflammation, scarring, and tangential illumination [34,35]. Although we demonstrated that the fluorescent signal could be indirectly confirmed with FECH staining, improved specificity may be further explored by enhancing the device, potentially via alternative fluorescence channels or multispectral sensing. Notably, the microendoscopic system developed in this study is well-suited for adaptation to other fluorophores and other imaging targets. Changing the excitation and emission filters could be sufficient to target different visual fluorophores, and switching the light source could make near-infrared fluorophores accessible, such as indocyanine green.

In future work, we plan to demonstrate the system clinically for the fluorescence sensing of bladder cancer. The present device was well suited for comparing the clinical significance of FDA-approved visible-region fluorescent contrast agents, such as 5-ALA with near-infrared phosphors such as indocyanine green, and had a structure that enabled it to acquire fluorescence signals through insertion into the biopsy channel of clinical devices, such as cystoscopes or bladder surgical robots.

## 5. Conclusions

For minimally invasive bladder brightfield imaging and fluorescence sensing in small animals, an optical system was constructed based on a 1.2 mm diameter microendoscope. Specifically, 360–440 nm optical and 610–690 emission filters were mounted for PpIX fluorescence excitation and sensing, respectively. A small-diameter image fiber bundle magnified component and a 1080-pixel high-resolution camera were coupled to the system to allow in vivo preclinical studies. The diagnostic potential and applicability of this microendoscopic fluorescence sensing system were evaluated in a carcinogen-induced animal model, and brightfield and fluorescence imaging inside the bladder was recorded and analyzed every three weeks. The time of bladder cancer occurrence varied between 7 and 19 weeks, and the new detection system allowed us to monitor the bladder cancer development. For the in vivo bladder cancer tissue analysis, 5-ALA was orally administered, and fluorescence was sensed based on the accumulation of PpIX in the cancer tissue. The cancer detection capability and sensitivity of this microendoscopic fluorescence sensing system were further verified with a morphological analysis and FECH staining, which strongly correlated with PpIX metabolism.

## Data Availability

Original and raw data files are available from the authors upon reasonable request.
